# Data on the femtosecond laser-induced formation of tracks in silver-containing zinc phosphate glass

**DOI:** 10.1016/j.dib.2020.106698

**Published:** 2020-12-26

**Authors:** Georgiy Shakhgildyan, Alexey Lipatiev, Maxim Vetchinnikov, Sergey Fedotov, Sergey Lotarev, Vladimir Sigaev

**Affiliations:** Mendeleev University of Chemical Technology, Moscow, Russia

**Keywords:** Phosphate glass, Direct laser writing, Silver nanoparticles, Silver clusters

## Abstract

Direct femtosecond laser writing of tracks in the volume of silver-containing zinc phosphate glass is investigated. Tracks were written by the femtosecond laser irradiation with the wavelength of 1030 nm, the pulse duration of 180 fs and a scanning speed of 1 mm/s. The data shows the effect of the pulse repetition rate (10, 100 and 500 kHz) and the pulse energy (60–120 nJ) on the microstructure and optical properties of the laser-written tracks. The data was collected by optical microscopy in the brightfield and fluorescence mode. Moreover, the data shows the dependence of the width and height of the laser-written tracks on the pulse energy and pulse repetition rate.

## Specifications Table

**Table:** 

Subject	Material Science Engineering
Specific subject area	Laser material processing
Type of data	Image
	Graph
	Figure
How data were acquired	Optical brightfield (BF) and fluorescence (FL) microscopy (OM; Olympus BX51)
Data format	Analyzed
Parameters for data collection	Silver-containing phosphate glass sample with the optically polished surfaces after exposure to the scanning femtosecond laser beam was used to perform optical microscopy study at room temperature. The study was conducted in the two planes: perpendicular to laser beam writing (top-view) and the beam propagation direction (cross-section view).
Description of data collection	Effects of the femtosecond laser exposure parameters on the microstructure and optical properties of the silver-containing phosphate glass are demonstrated via processed optical microscopy data.
Data source location	Dr. G. Yu. Shakhgildyan, Mendeleev University of Chemical Technology, Moscow, Russia
Data accessibility	Repository name: Mendeley Data
	Data identification number: 10.17632/dhy5yr95kd.1
	Direct URL to the data: http://dx.doi.org/10.17632/dhy5yr95kd.1

## Value of the Data

• The data shows the changes in the microstructure of the tracks laser-written in the silver-containing phosphate glass at different incident laser pulse energy and pulse repetition rate.
• The data will contribute to the understanding of the thermally driven processes during the formation of tracks in glass induced by laser irradiation.
• The data could be used for experimenters and as basic data for further research in the field of direct laser writing in the silver-containing glasses and the development of the nonlinear optical waveguides.

## 1 Data Description

**Fig. 1:**
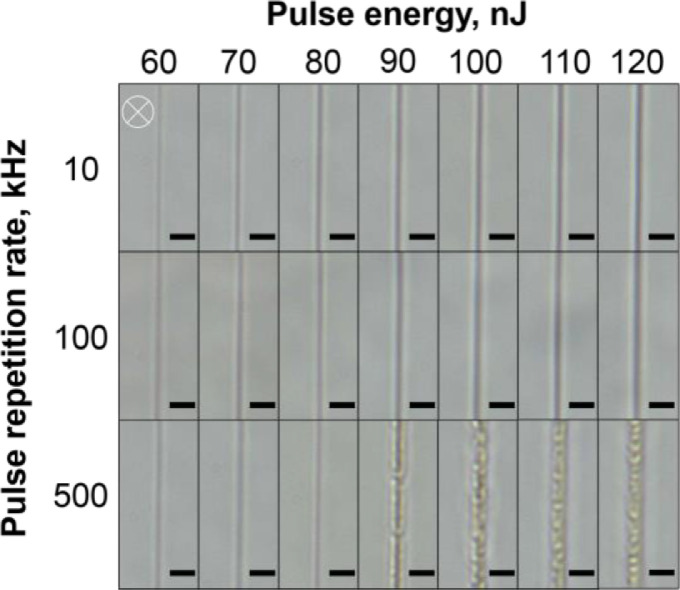
Top-view BF microscope images of the silver-containing phosphate glass after laser irradiation with different incident laser pulse energy and pulse repetition rate. The scanning speed was 1 mm/s. The white crosshair indicates a laser beam propagation direction. A scale bar is 5 μm.

The optical images of the tracks written in the silver-containing phosphate glass at different laser pulse energy and pulse repetition rate shown in Fig. 1 are obtained by the optical microscopy in brightfield (BF) mode. The tracks are formed by translating the glass sample in a direction perpendicular to the propagation direction of the focused laser beam with the scanning speed of 1 mm/s.

**Fig. 2:**
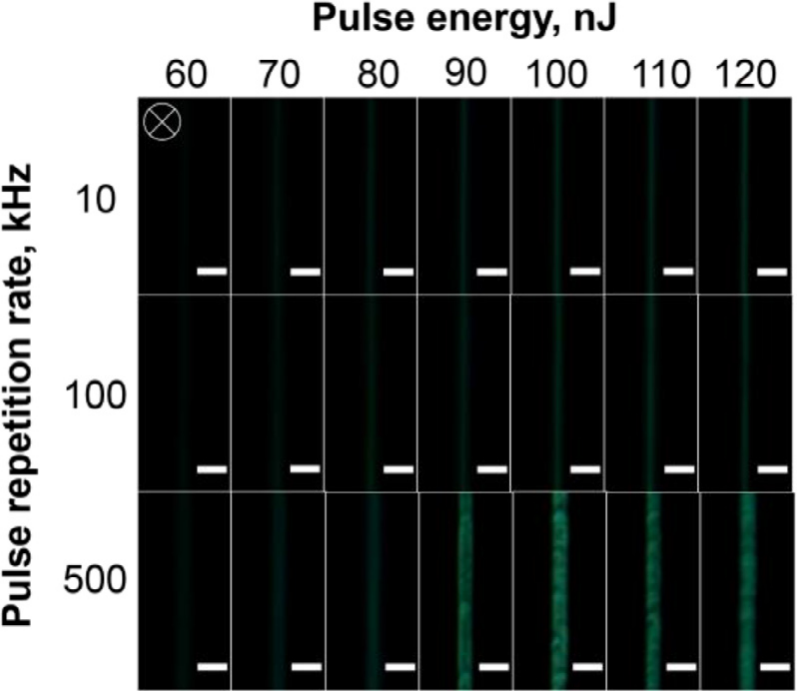
Top-view FL microscope images of the silver-containing phosphate glass sample after laser irradiation at different incident laser pulse energy and pulse repetition rate. The scanning speed was 1 mm/s. The white crosshair indicates a laser beam propagation direction. A scale bar is 5 μm.

Fig. 2 presents the optical microscopy images of the tracks written in silver-containing phosphate glass in fluorescence (FL) mode under the excitation in the range of 400–410 nm and registered in the 455–800 nm spectral range.

**Fig. 3:**
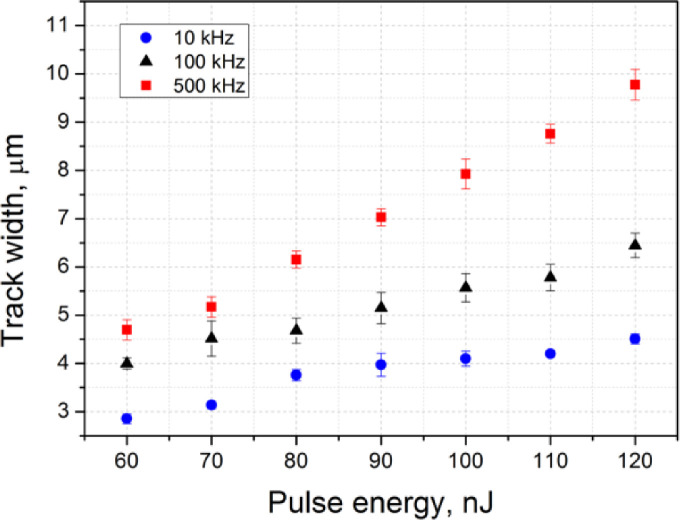
The dependence of the laser-written track width on the incident laser pulse energy for three different pulse repetition rates.

Fig. 3 presents the dependence of the laser-written track width on the writing laser pulse energy for three different pulse repetition rates (10, 100 and 500 kHz). The raw data for Figs. 3 and 6 are provided in the Mendeley dataset.

**Fig. 4:**
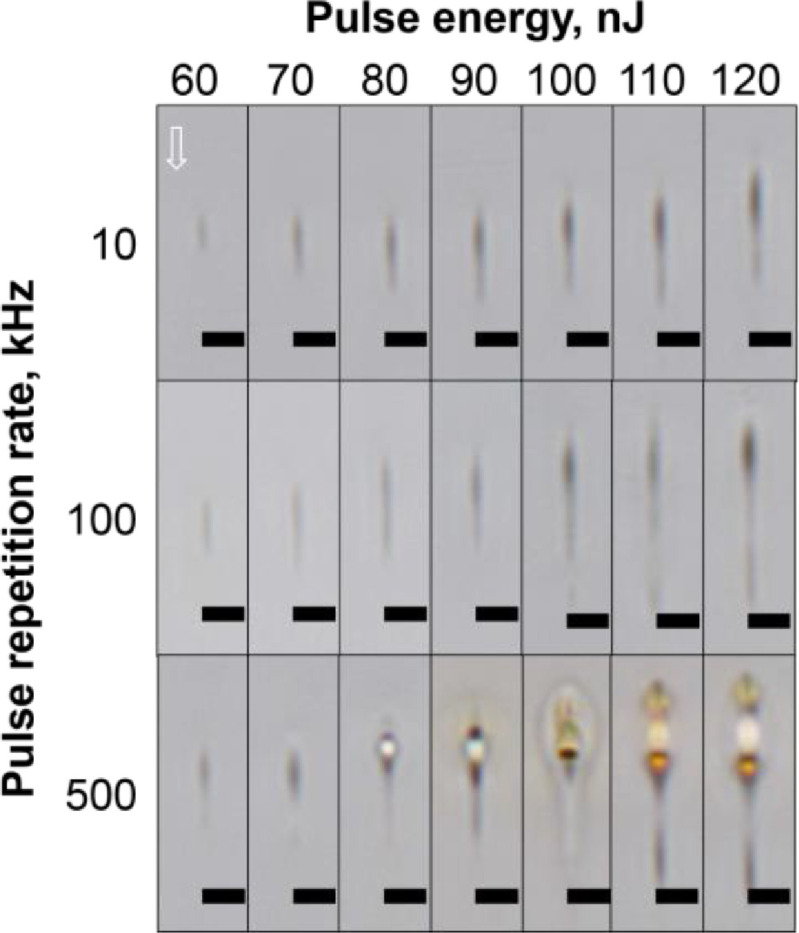
Cross-section view BF microscope images of the tracks written in the silver-containing phosphate glass sample at different incident laser pulse energy and pulse repetition rate. The scanning speed was 1 mm/s. The white arrow indicates the direction of the laser beam propagation. The scan was directed to the observed side. A scale bar is 5 μm.

The cross-section view of the tracks written in the silver-containing phosphate glass with different laser pulse energy and pulse repetition rates are shown in Fig. 4 with the optical microscopy images in the BF mode.

**Fig. 5:**
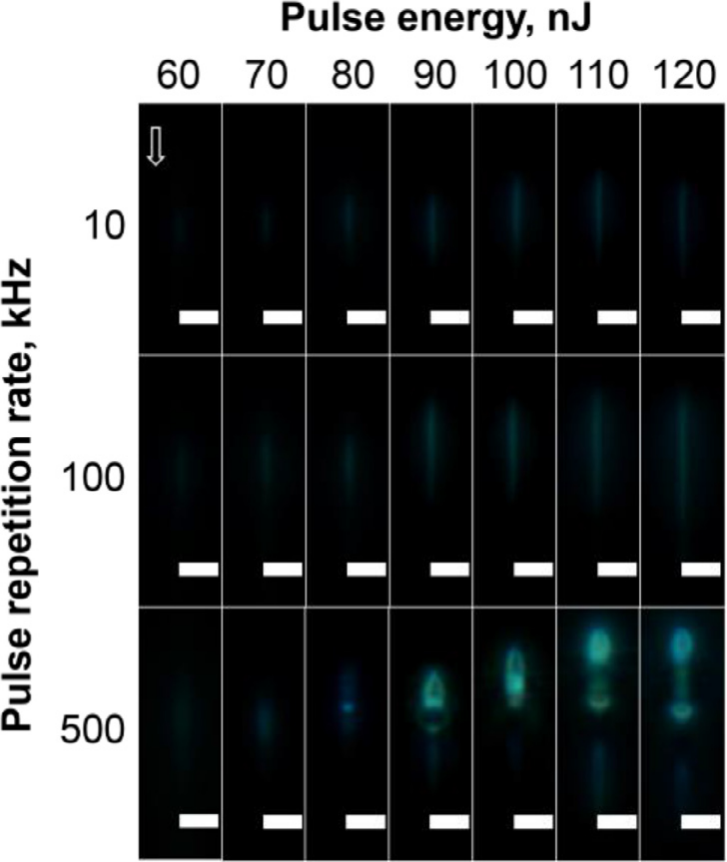
Cross-section view FL microscope images of the tracks written in the silver-containing phosphate glass sample at different incident laser pulse energy and pulse repetition rate. The scanning speed was 1 mm/s. The white arrow indicates the direction of the laser beam propagation. The scan was directed to the observed side. A scale bar is 5 μm.

The cross-section view of the tracks written in the silver-containing phosphate glass with different laser pulse energy and pulse repetition rates with the optical microscopy images in the FL mode under the excitation in 400–410 nm and registration in a 455–800 nm spectral range are shown in Fig. 5.

**Fig. 6:**
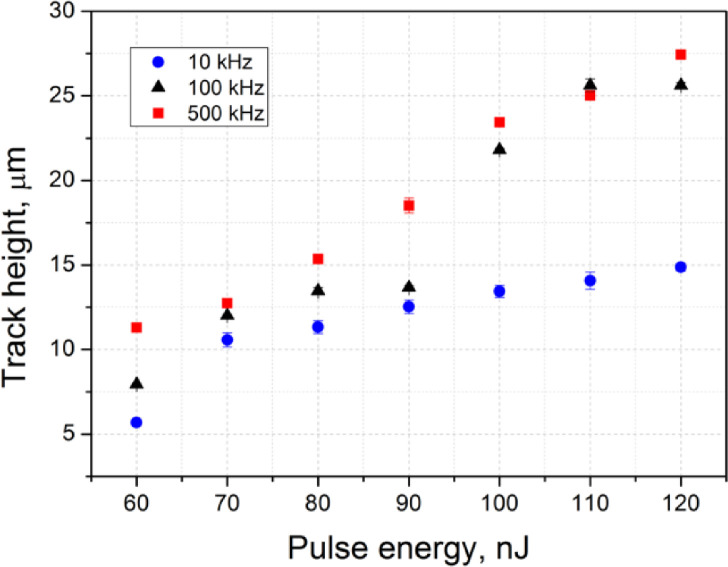
The dependence of the laser-written track height on the incident laser pulse energy for three different pulse repetition rates.

Fig. 6 shows the dependence of the laser-written track height on the writing laser pulse energy for three different pulse repetition rates (10, 100 and 500 kHz).

## 2 Experimental Design, Materials and Methods

### 2.1 Experimental design

A Yb:KGW femtosecond laser system Pharos SP (Light Conversion Ltd.) with a regenerative amplifier operating at wavelength λ = 1030 nm was used for writing tracks in the glass sample. The repetition rate of laser pulses was 10, 100 and 500 kHz. Pulse duration was 180 fs. The energy of the pulses entering the sample was controlled with the motorized polarization attenuator and changed from 60 to 120 nJ. A motorized half-wave plate controlled the direction of linear polarization of the laser beam. The plane of the laser polarization was parallel to the laser beam scanning direction. The laser beam was focused in the glass plate volume with an objective lens with a numerical aperture NA = 0.65 (Olympus LCPLN50XIR) at a depth of 150 μm. A spacing of 200 μm between tracks is used to avoid overlapping. The plate of glass with the size of 0.4 × 1.5 × 2.5 cm was used for tracks writing. The glass sample was set on the air-bearing stage (Aerotech ABL1000) synchronized with the laser by SCA Professor software, which allowed 3D precise positioning of the glass sample with the 1 mm/s scanning speed.

### 2.2 Materials

Silver-containing zinc phosphate glass with the following composition: 8Ag20⋅53ZnO⋅39 P_2_O_5_ (mol%) was used. Glass was obtained by a melt-quenching technique using high-purity precursors (AgNO_3_, ZnO, H_3_PO_4_), which were mixed and melted at 1200 °C in a corundum crucible covered with the fused silica cap for 2 h. After that, glass melt was rapidly quenched in a preheated metal form and annealed at 325 °C for 4 h to relax mechanical stress. Detailed description of glass synthesis has been given elsewhere [1], [2]. Composition of the glass batch was calculated for the production of 150 g glass block in order to achieve optically homogeneous glass free of striae. The glass block was cut and polished to optical quality plates for the laser treatment experiments. For the further characterization of the written tracks, the facets of the glass sample were optically polished.

### 2.3 Methods

To study the structure of the laser-written tracks, optical images of the tracks in the brightfield (BF) and fluorescence (FL) modes were obtained using an Olympus BX51 microscope equipped with an Olympus DP73 CCD camera. The fluorescence excitation was provided by the mercury lamp and the interference filter with the transmission band at 400–410 nm. The emission was registered in the 455–800 nm spectral range. All the FL images taken in the top-view configuration were recorded with the 200 ms exposure and all the cross-section view images were recorded with the 100 ms exposure. All the obtained images were processed using the ImageJ software [3]. The dependence of the track width (Fig. 3) and the track height (Fig. 6) was obtained using the measured dimensions from Figs. 1 and 4.

## CRediT Author Statement

**Georgiy Shakhgildyan**: Writing - Original Draft, Visualization; **Alexey Lipatiev**: Investigation, Writing - Review & Editing; **Maxim Vetchinnikov**: Investigation, Validation; **Sergey Fedotov**: Investigation; **Sergey Lotarev**: Supervision, Writing - Review & Editing; **Vladimir Sigaev**: Conceptualization, Resources.

## Declaration of Competing Interest

The authors declare that they have no known competing financial interests or personal relationships which have, or could be perceived to have, influenced the work reported in this article.
